# Biochemical Profile of the Soybean Seed Embryonic Axis and Its Changes during Accelerated Aging

**DOI:** 10.3390/biology9080186

**Published:** 2020-07-23

**Authors:** Luciano Antônio Ebone, Andréia Caverzan, Diógenes Cecchin Silveira, Luciano de Oliveira Siqueira, Nadia Canali Lângaro, José Luís Trevizan Chiomento, Geraldo Chavarria

**Affiliations:** 1 Laboratory of Plant Physiology, Agronomy Post-Graduate Program, University of Passo Fundo, BR 285 Km 171, Passo Fundo, Rio Grande do Sul 99052-900, Brazil; lucianoebone9@gmail.com (L.A.E.); acaverzan@hotmail.com (A.C.); 2 Departament of Forage Plant and Agrometeorology, Animal Science Post-Graduate Program, Federal University of Rio Grande do Sul, Avenue Bento Gonçalves, 7712, Agronomia, Porto Alegre 91540-000, Brazil; diogenessilveira@hotmail.com; 3 Faculty of Pharmacy, Institute of Biological Sciences, University of Passo Fundo, BR 285 Km 171, Passo Fundo, Rio Grande do Sul 99052-900, Brazil; luciano@upf.br; 4 Laboratory of Seed Technology, Agronomy Post-Graduate Program, University of Passo Fundo, BR 285 Km 171, Passo Fundo, Rio Grande do Sul 99052-900, Brazil; nclangaro@upf.br; 5 Laboratory of Olericulture, Agronomy Post-Graduate Program, University of Passo Fundo, BR 285 Km 171, Passo Fundo, Rio Grande do Sul 99052-900, Brazil; jose-trevizan@hotmail.com

**Keywords:** DNA damage, *Glycine max*, magnesium, Maillard reaction, lipid peroxidation, vigor, seed deterioration

## Abstract

Seed deterioration is an important topic in plant science, as the majority of cultivated species use seeds as their means of propagation; however, due to its complexity, the process of seed deterioration has not yet been completely elucidated. Three soybean cultivars (BMX Raio, BMX Zeus, and DM 53i54) exposed to four distinct periods of accelerated aging (0, 3, 6 and 9 days) in a fully randomized experimental design. Initially, vigor and germination tests were performed. The activity of superoxide dismutase, catalase, ascorbate peroxidase enzymes, hydrogen peroxide, malonaldehyde, DNA oxidation, macromolecules and mineral content, and Maillard reactions were quantified in the embryonic axis. Results showed that DNA did not suffer degradation or oxidation. In terms of consumption of reserves, only sugars were consumed, while levels of protein, starch, and triglycerides were maintained. The Maillard reaction did show potential as an indicator of buffer capacity of protein to ROS. Additionally, levels of catalase and ascorbate peroxidase decreased during the aging process. Moreover, nutrient analysis showed that a high magnesium level in the cultivar bestowed greater resilience to deterioration, which can indicate a potential function of magnesium in the cell structure via reflex in seed aging through seed respiration.

## 1. Introduction

Seed deterioration is an important topic in plant science, as seed is the means of propagation for the majority of cultivated species [1]; in addition, seed deterioration has not yet been completely elucidated due the complexity of this phenomenon [2]. Nevertheless, it is known that deterioration leads to a reduction of seed quality, which, in turn, causes a reduction in crop yield [3,4]. In a world where population levels and food demand are increasing, it is imperative to improve our understanding of the seed deterioration process in order to take measures to avoid its occurrence, thereby helping to improve crop yield and ensuring food security.

Accordingly, it is necessary to examine the main deterioration processes happening in the seed—such as inactivation of antioxidant enzymes, membrane damage, reserve consumption, and material genetics damage [5]—so that a robust response to the deterioration mechanism can be determined. The first event in seed aging is the depression of antioxidant enzymes such as superoxide dismutase (SOD), catalase (CAT), ascorbate peroxidase (APX), and glutathione peroxidase (GPX) [5]. The depression of the antioxidant enzyme system is caused by the down-regulation of and reduction in scavenging antioxidant activity [6]. This imbalance of the antioxidant system leads to the accumulation of reactive oxygen species (ROS), which is mainly hydrogen peroxide (H_2_O_2_) due to its relatively long half-life (1 ms) compared to the other forms of ROS (2–4 µs) [7].

The imbalance in antioxidant activity versus high ROS production generates damage to cell membrane and genetic material [8]. Genetic damage occurs by the oxidation of the bases of DNA and RNA causing chromosome aberrations, DNA double-strand breaks, growth inhibition, and loss of viability [9]. Membrane damage caused by lipid peroxidation is the main event in seed deterioration that changes cell permeability [8], since the reaction of lipids with ROS lead to the production of compounds with distinct solubility, such as malondialdehyde (MDA) [10].

The embryo is responsible for the origination of the new seedling; however, very few works measure this tissue, and those that do either only englobe a small portion of each deterioration process [11,12,13], or they investigated deterioration in the seed as whole (embryo and endosperm) [10]. Therefore, many questions still remain today about seed deterioration and embryonic axis tissue. To wit, what are the concentrations of sugars, starch, proteins, macronutrients, and other components in the embryonic axis of soybean (*Glycine max* [L.] Merril); how do these concentrations impact resilience to deterioration; and are the events that happen in the whole seed equal to what happens in the embryo alone.

The hypothesis of this work was developed by Ebone et al. [5], where the deterioration process occurs in three phases. In the first phase is the beginning of deterioration, with a slight reduction of vigor caused by the reaction of reducing sugars with antioxidant enzymes and genetic material. In the second phase, the cell presents oxidative damage causing lipid peroxidation, which leads to leaching of solutes, the formation of malondialdehyde, and, consequently, an increase in the damage to the genetic material. In the third phase, there is cell collapse, with mitochondrial membrane deconstruction and high accumulation of ROS, malondialdehyde, and reducing sugars. The objective of the study was to evaluate the macroconstituents and characters linked to the deterioration of the embryonic axis of soybean seeds, in different periods of accelerated aging and cultivars.

## 2. Materials and Methods

### 2.1. Plant Material, Accelerated Aging, Germination, and Vigor Tests

The plant material used in the experiment comprised three cultivars of soybean (BMX Raio RSF IPRO, BMX Zeus RSF IPRO, and DM 53i54 RSF IPRO). The seeds were produced in a commercial field with humid, subtropical climate region with humic dystrophic Red Latosol, with oat (*Avena strigosa* Schreb) as the previous crop. After harvest seeds were processed in seed processing unit, then 1 kg of each cultivar was collected. Seeds were placed in Bio-Oxygen Demand Incubator (BOD) in the dark and exposed to 32 °C heat and 95% humidity [3], for 0, 3, 6, and 9 days. Initially, germination and vigor tests were performed in seeds. Germination was determined with four samples of 100 seeds, each put in wrapped, wet germination paper, which were placed in a Mangelsdorf-type germination chamber and maintained at 25 °C for seven days, being the number of normal seedlings read at the end of this period [14]. For the vigor test, seeds were exposed at 42 °C for two days in aging chambers in the dark, and then the germination procedure was followed [15].

### 2.2. Isolation of the Soybean Embryonic Axis

For the isolation of the embryonic axis, seeds had their coat removed and embryo excised, and were immediately frozen in N_2_ liquid. Next, samples were kept in a freezer at −80 °C until analysis was conducted.

### 2.3. Biochemical Analyses

For protein, glucose, cholesterol, triglycerides, lipid peroxidation, hydrogen peroxide, Maillard reaction, and enzymatic activity analyses, approximately 200 mg of tissue (soybean embryo axis) was macerated in 3.8 mL of potassium phosphate buffer 100 mM (pH 7.0).

#### 2.3.1. Antioxidant Enzymes

The reaction of ascorbate peroxidase (APX) was started by adding 30 mM H_2_O_2_. The enzyme activity was measured by the decrease in absorbance at 290 nm, 25 °C, for 300 s [16]. To avoid interference by type III peroxidase activity, two parallel determinations were performed: (A) in the absence or (B) in the presence of p-Chloromercuribenzoic acid (pCMB), a specific APX inhibitor [17]. The net APX activity was calculated from the difference, A–B, and it was expressed as μmol AsA mg^−1^ protein^−1^·min^−1^.

CAT activity was measured by following the oxidation of H_2_O_2_ at 240 nm, and was determined from the reaction of the crude extract in the presence of 50 mM potassium phosphate buffer (pH 7.0) containing 20 mM H_2_O_2_. The reaction occurred at 30 °C, and the absorbance was monitored at 240 nm for 300 s [18]. CAT activity was calculated according to the molar extinction coefficient of H_2_O_2_ (36 mM^−1^ cm^−1^) and expressed as μmol H_2_O_2_ mg protein^−1^ min^−1^.

SOD activity was determined by the inhibition of blue formazan production by NBT photoreduction, and was measured by adding embryo extract to a mixture containing 50 mM K-phosphate buffer (pH 7.8), 0.1 mM EDTA, 13 mM L14 methionine, 2 M riboflavin, and 75 M p-nitro blue tetrazolium chloride (NBT) in the dark. The reaction was carried out under illumination (30-watt fluorescent lamp) at 25 °C for 6 min. Absorbance was measured at 540 nm [19]. One SOD activity unit (U) was defined as the amount of enzyme required to inhibit 50% of the NBT photoreduction. Quantification enzymes were performed in technical and biological triplicate.

#### 2.3.2. Lipid Peroxidation, Hydrogen Peroxide, and Maillard Reaction

Lipid peroxidation was measured using thiobarbituric acid-reactive substances (TBARS) [20]. The concentration of TBARS was calculated using the absorption coefficient of 155 mM^−1^ cm^−1^, and the results were expressed as µmol MDA TBA g FW^−1^. Malondialdehyde was performed in technical and biological triplicate.

Hydrogen peroxide (H_2_O_2_) content was measured by the titanium tetrachloride method [21]. The storage solution had 5% (*w*/*v*) of trichloroacetic acid added and was then centrifuged at 12,000× *g* (4 °C), and the supernatant was immediately used for H_2_O_2_ determination. Measurement was performed after the reaction of the titanium reagent with H_2_O_2_ and the formation of the hydroperoxide-titanium complex. H_2_O_2_ content was calculated from a standard curve and read at 415 nm. Results were expressed as µmol H_2_O_2_ g^−1^ FW. Hydrogen peroxide was performed in technical and biological triplicate.

For the Maillard reaction, 0.15 mL of the extract of soybean embryo axis was filtered through a Millipore Millex-HA 0.45 filter. The Maillard product was then measured with fluorescence measured at 440 nm using a fluorescence spectrophotometer with excitation at 370 nm [11].

#### 2.3.3. Protein, Glucose, Cholesterol, Triglycerides, Starch, and Sugars

Biochemical dosages of glucose, proteins, triglycerides, total cholesterol, and fractions were determined using Labtest Diagnóstica^®^ kits. For all measures, specific protocols were used according to the manufacturer’s guidelines. Concentrations were determined using semiautomatic equipment (Biosystems BTS 350, Barcelona, Spain). All evaluations were made in triplicate.

For the determination of sugar and starch content, embryonic axes were dried in air at 60 °C until constant mass. Total soluble sugar content was then determined by the phenolsulfuric acid method [22]. The resulting pellet was treated with perchloric acid, and aliquots of the supernatant were used to determine the starch content [23]. The experiment was performed in triplicate, and the results are expressed as mmol kg^−1^ dry mass (mmol kg^−1^ DM).

### 2.4. Macronutrients Analysis

Nitrogen (N), phosphorus (P), potassium (K), calcium (Ca), and magnesium (Mg) content was determined using the method described by Tedesco et al. [24]. For this, 0.25 g of dry embryonic axis was ground and then submitted to digestion with addiction of 1 mL of H_2_O_2_ (0.4 M) + 2 mL of H_2_SO_4_ (0.4 M) + 0.7 g of solution 20 mM (Na_2_SO_4_ + CuSO_4_ + Se). The samples were taken to the digester block and kept at 180 °C degrees until the water evaporated, after the temperature was increased to 350 °C until the sample reached greenish-yellow color, remained in the digestor block for another hour after reaching the color. After digestion, the samples were removed from the digester block, cooled, and added distilled and deionized water until it reached 50 mL. The concentration of nitrogen was determined using the Kjeldahl method, the phosphorus concentration was determined by spectrophotometry, potassium by flame photometry, calcium and magnesium by atomic absorption spectrophotometry [24].

### 2.5. Extraction and Estimation Analysis, DNA Oxidation

Total DNA of soybean embryonic axis was isolated following the procedure of Doyle and Doyle [25], which 500 mg of tissue was macerated in liquid nitrogen and added to CTAB buffer, incubated at 60 °C for 60 min. DNA was extracted with chloroform-isoamyl alcohol (24:1) and precipitated with cold isopropanol. The DNA isolated was washed with wash buffer and resuspended in TE buffer, and stored at −20 °C [25]. Both quality and quantity of isolated DNA were assessed spectrophotometrically (260/280 nm) and by electrophoresis 0.8% (*w*/*v*) agarose gel, and residual DNA was stored in at −20 °C.

DNA oxidation was quantified in two replicates following the method of Langfinger and Von Sonntag [26]. For this, 20 µL of isolated DNA (1.75–1.8 purity) was mixed with 2 mL of 0.6% (*w*/*v*) thiobarbituric acid. Samples were heated at 90 °C for 30 min, and were then measured at 537 nm.

### 2.6. Scanning Electron Microscopy (SEM)

Soybean embryonic axes were observed by scanning electron microscope using the technique of Robards [27]. Samples were fixed in 2.5% glutaraldehyde, 4.0% paraformaldehyde, and a 0.05 M sodium cacodylate buffer (pH 7.2). Next, samples were post-fixed in 1% osmium tetroxide in the same buffer for 1 h, and dehydrated in an alcoholic series. The material was subsequently submitted to the critical point of CO_2_ and covered with a 3-nm layer of gold. Samples were observed under a scanning electron microscope (Tescan, Vega LM 3) operated at 20 Kv to obtain electromicrographs.

### 2.7. Statistical Analysis

Analysis of variance (ANOVA) was used, considering the factors of treatment cultivar and time with a completely randomized design, and in case of significant difference, the Tukey test was applied at 5% probability of error for comparisons between means. Normality and homogeneity as well as variances of data were verified by the Shapiro–Wilk test and by the Bartlett test, with no need to transform the data into the variables under study.

Pearson’s correlation coefficients between the variables studied were estimated, whose significance was assessed by Student’s t-test, at 1% and 5% probabilities. To graphically express the functional relationship between the correlation between the characteristics, a correlation network in which the proximity between the features was proportional to the absolute value between their correlations was used. The thickness of the edges was controlled by applying a cutoff value of 0.60, which meant that only ≥0.60 had their edges highlighted. Positive correlations were represented in blue, while negative correlations were represented in yellow. When an association between the variables was verified, trail analysis was performed, considering separately the basic variables (main dependent) of germination (GER) and vigor (VIG), whereas SOD, CAT, APX, H_2_O_2_, malondialdehyde (MAL), Maillard reaction (MAI), N, P, K, Ca, and Mg served as explanatory variables.

Subsequently, multivariate analysis was performed by generating the Mahalanobis distance matrix (D2). Then, it was tested by means of cophenetic correlation analysis (r = 0.73) in order to determine which of the hierarchical grouping methods expressed the best fit, resulting in the choice of the Unweighted Pair Group Method Arithmetic Average (UPGMA). The number of groups was defined by the Mojena [28] procedure, which proposes a calculation procedure based on the relative size of dendrogram distances.

## 3. Results

### 3.1. Germination and Vigor under Accelerated Aging

**Figure 1:**
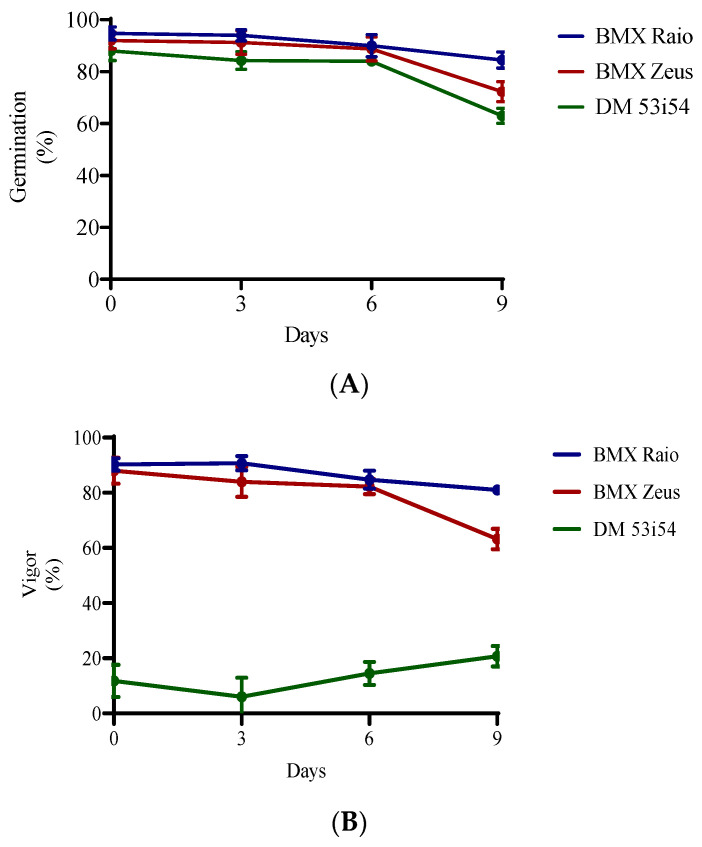
Germination (**A**) and vigor (**B**) of three cultivars (BMX Raio, BMX Zeus, and DM 53i54) exposed to different periods of accelerated aging (0, 3, 6, and 9 days).

The germination of cultivars not exposed to deterioration (0 days) were 95% for cultivar BMX Raio, 92% for BMX Zeus, and 88% for DM 53i54. All cultivars had reductions in their germination percentage of less than 5% until 6 days accelerated aging. Significant reductions in germination occurred at 9 days, at which point germination decreased to 85%, 72%, and 63% for BMX Raio, BMX Zeus, and DM 53i54, respectively (Figure 1). For the vigor variable, the cultivars BMX Raio (90.2%, 90.7%, 84.7%, and 81.0% at 0, 3, 6, and 9 days, respectively) and BMX Zeus (88.0%, 84.0%, 82.2%, and 63.2% at 0, 3, 6, and 9 days, respectively) showed similar values until 9 days, when the cultivar BMX Zeus showed a greater reduction (−19%) in this variable than the cultivar BMX Raio (−3.7%) when compared at 6 and 9 days. The cultivar DM 53i54 showed low vigor values from day 0 and erratic behavior throughout accelerated aging, with a reduction at 3 days, but an increase at 6 and 9 days.

### 3.2. Antioxidant Enzyme Activity

**Figure 2:**
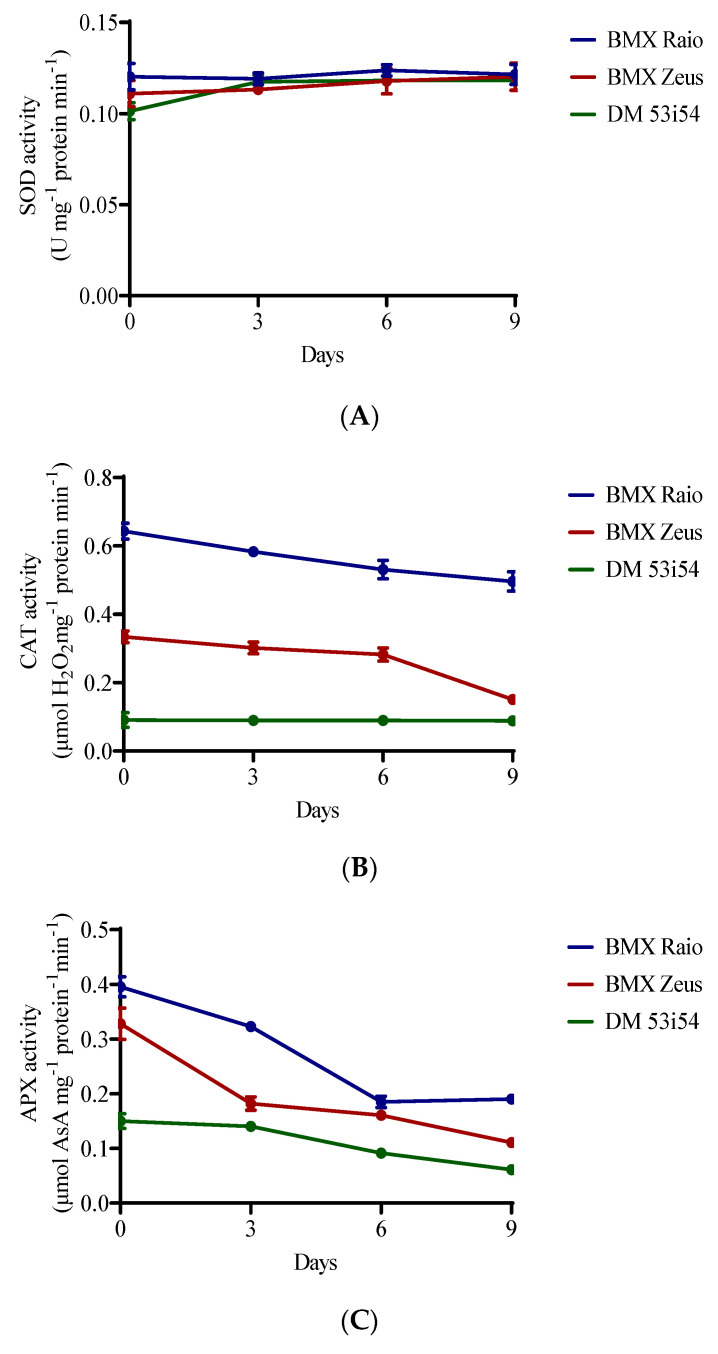
Enzymatic activity of superoxide dismutase (SOD) (**A**), catalase (CAT) (**B**), and ascorbate peroxidase (APX) (**C**) of soybean seed embryonic axis of three cultivars (BMX Raio, BMX Zeus, and DM 53i54) exposed to different periods of accelerated aging (0, 3, 6, and 9 days).

SOD activity showed resilience to the studied accelerated aging period, remaining stable across cultivars and with minor changes through aging periods. A different behavior was presented for CAT and APX enzymes, which showed differences between cultivars and between periods of accelerated aging (Figure 2). The cultivar BMX Raio had a superior content to that of BMX Zeus, which was itself superior to DM 53i54 for CAT and APX. BMX Raio maintained values of CAT content that were 1.9 times greater than those for BMX Zeus until 9 days, when the ratio becomes 3.3 times greater. In this sense, the decrease in CAT activity in BMX Zeus at 9 days was 46%, which was marked by a 19% reduction in vigor level. The cultivar DM 53i54 did not show a reduction of CAT through aging; however, its content was low. For APX, the main reduction for cultivars BMX Raio and DM 53i54 occurred at 6 days, and for the cultivar BMX Zeus, at 3 days; thus, all cultivars showed changes in this variable during aging.

### 3.3. Hydrogen Peroxide, Lipid Peroxidation, and Maillard Reaction

**Figure 3:**
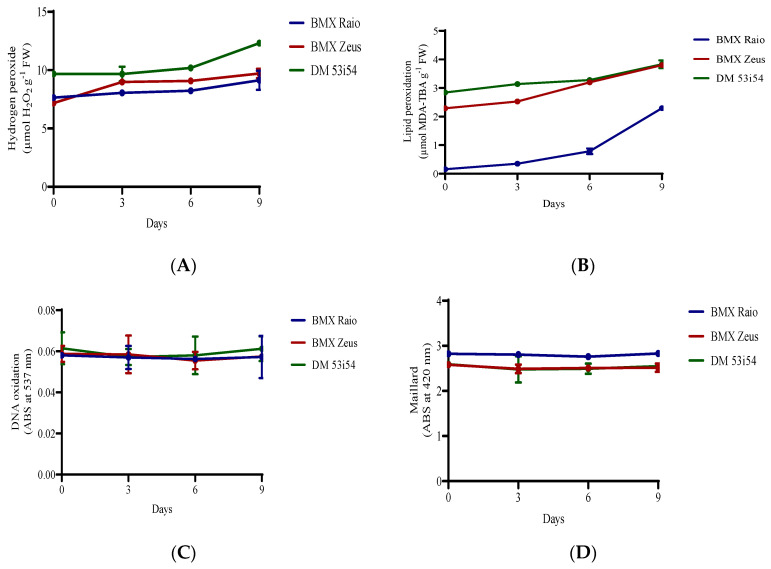
H_2_O_2_ (**A**) and malondialdehyde (**B**) content, DNA oxidation (**C**), and Maillard reaction (**D**) of soybean seed axis of three cultivars (BMX Raio, BMX Zeus, and DM 53i54) exposed to different periods of accelerated aging (0, 3, 6, and 9 days).

The cultivar DM 53i54 showed a high content of H_2_O_2_ (9.6 μmol g^−1^ FM) at 0 day (Figure 3); it closely maintained this value until 9 days, when the level of H_2_O_2_ increased 28% (12.3 μmol g^−1^ FM). At 0 day, the cultivar BMX Raio (7.6 μmol g^−1^ FM) showed a content higher than that of BMX Zeus (7.2 μmol g^−1^ FM). However, during the accelerated aging at 3 days, the cultivar BMX Raio showed an increase of 5.4%, while the cultivar BMX Zeus in that same period showed an increase of 25.2%, leading to higher values of H_2_O_2_ content for the cultivar BMX Zeus than for BMX Raio. The cultivar BMX Raio showed a lower MDA than the other cultivars, starting with only 0.15 μmol g^−1^ FM at 0 day and ending with 2.29 μmol g^−1^ FM at 9 days, a value equal to that showed by cultivar BMX Zeus at 0 day. The cultivar DM 53i54 showed higher concentrations of MDA at 0 day, but had a small increase, leading to the cultivar BMX Zeus reaching their values at 6 and 9 days. Values of DNA oxidation did not show differences among cultivars and periods of accelerated aging. In addition, the Maillard reaction did not show differences through periods of accelerated aging, but it did show distinction among cultivars, with higher values found for the cultivar BMX Raio.

**Figure 4:**
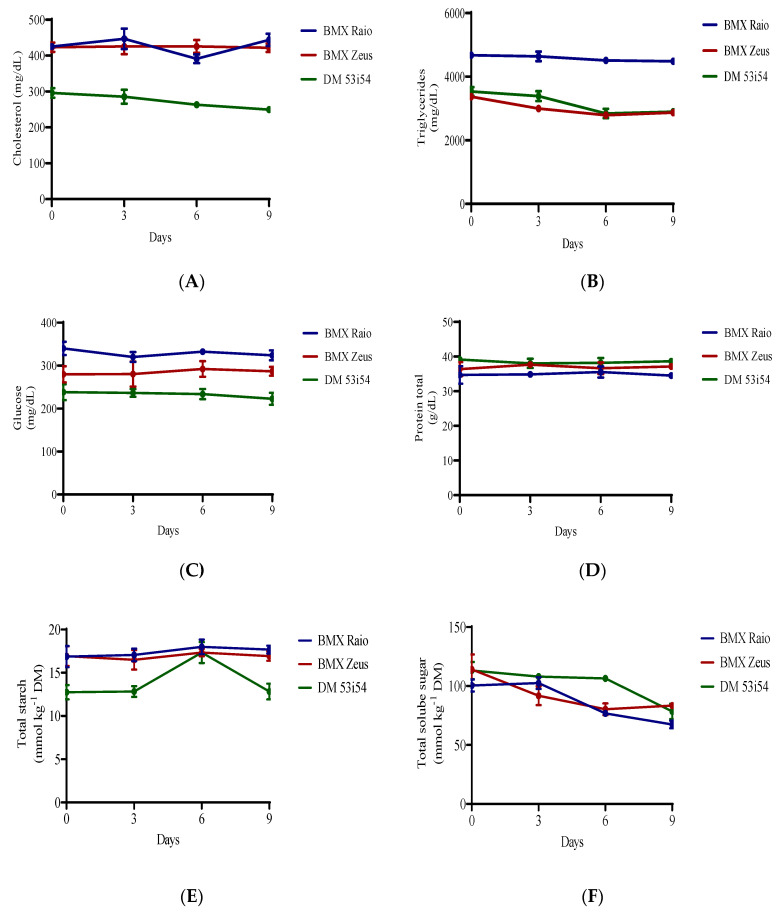
Cholesterol (**A**), triglycerides (**B**), glucose (**C**), protein (**D**), starch (**E**), and soluble sugars (**F**) content of soybean seed embryonic axis of three cultivars (BMX Raio, BMX Zeus, and DM 53i54) exposed to different periods of accelerated aging (0, 3, 6, and 9 days).

When analyzing the differences between the variables of cholesterol, triglycerides, protein, glucose, and starch, the periods of accelerated aging had significant influence only over cholesterol, starch and triglycerides, all the variables presented significant difference for cultivars (Figure 4). Sugar content, on the other hand, presented only significant differences among periods of accelerated aging with a clear and consistent reduction. In terms of sugar content, the cultivar BMX Raio initially showed the lowest reduction and BMX Zeus, the largest.

### 3.4. Macronutrients Analysis

**Figure 5:**
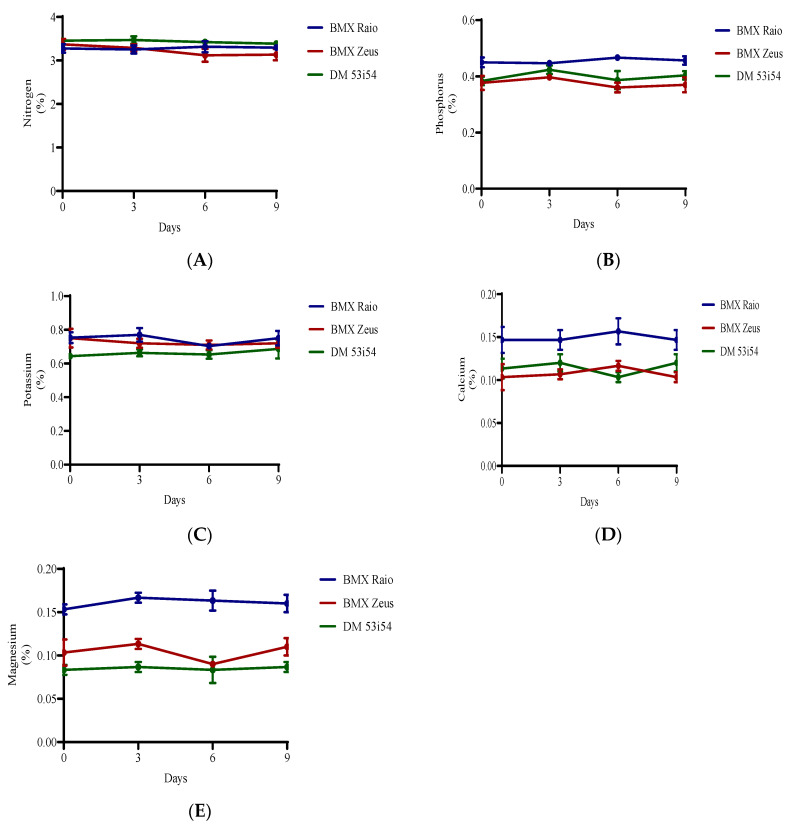
Macronutrients (nitrogen (**A**), phosphorus (**B**), potassium (**C**), calcium (**D**), and magnesium (**E**)) of soybean seed embryonic axis of three cultivars (BMX Raio, BMX Zeus, and DM 53i54) exposed (0, 3, 6 and 9 days).

Nitrogen was the most abundant macronutrient found in the soybean embryonic axis. The cultivar with the highest N levels was DM 53i54, followed by BMX Zeus and BMX Raio (Figure 5). In terms of protein:nitrogen ratios, the cultivars presented measures of 11.2, 11.5, and 10.6, respectively. For the variables Mg and K, the cultivar BMX Raio was superior, followed by BMX Zeus and DM 53i54.

### 3.5. Correlations

**Figure 6:**
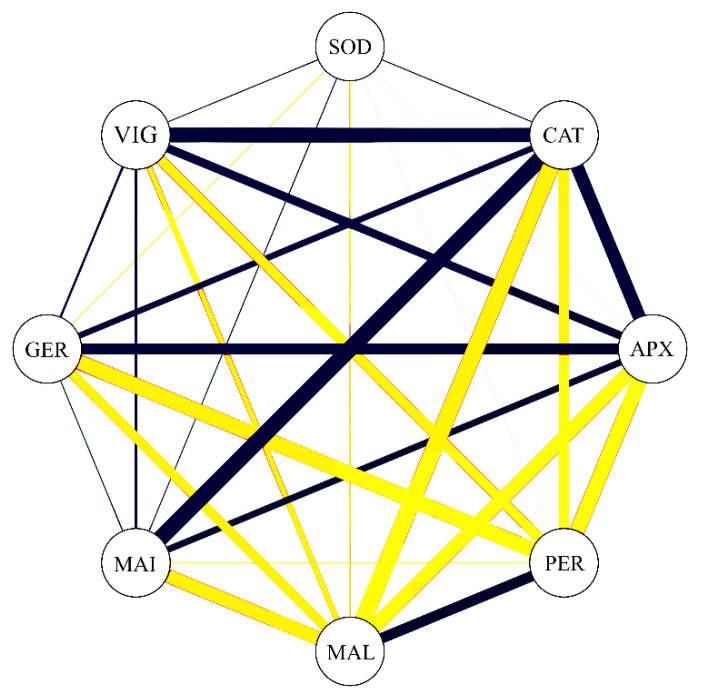
Correlation network among germination (GER), vigor (VIG), superoxide dismutase (SOD), catalase (CAT), ascorbate peroxidase (APX), hydrogen peroxidase (PER), malondialdehyde (MAL), and Maillard reaction (MAI). Blue lines indicate positive correlation, and yellow lines indicate negative correlation. Line thickness demonstrates the degree of association between the variables.

The correlation network showed that correlation coefficients between the studied enzymatic variables were represented, concomitantly with germination (GER) and vigor (VIG). Analyzing Figure 6, it is possible to verify that GER is positively correlated with CAT (0.63) and APX (0.74); negative correlations can be observed between the variables PER (−0.87) and MAL (−0.72). VIG showed the same association trend as GER; positive correlations were obtained between VIG and CAT (0.88) as well as APX (0.68), and negative correlations were obtained between VIG and PER (−0.74) as well as MAL (−0.62). These data confirm that the most important enzymes to increase germination and vigor in soybean seeds is APX and CAT. Otherwise, it can be inferred that PER, followed by MAL, contribute in an opposite way to the germination and vigor of soybean seeds.

**Figure 7:**
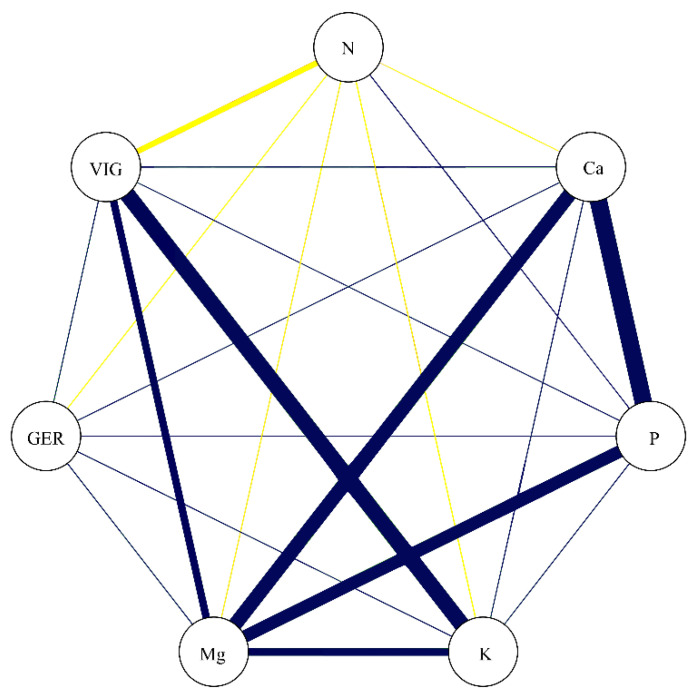
Correlation network among germination (GER), vigor (VIG), nitrogen (N), calcium (Ca), phosphorus (P), potassium (K), and magnesium (Mg). Blue lines indicate positive correlation, and yellow lines indicate negative correlation. Line thickness demonstrates the degree of association between the variables.

In the correlation network represented in Figure 7, the correlation coefficients between the studied nutrients are portrayed concomitantly with GER and VIG. While GER showed a lack of significant association with the studied nutrients, VIG presented a negative association with N (−0.69) and positive association with K (0.89) and Mg (0.72). These data confirm that the nutrients which most greatly contribute to increasing the vigor in soybean seeds are K and Mg.

Determination coefficients for GER (R^2^ = 0.87) and VIG (R^2^ = 0.75), which are associated with the residual effects (GER = 0.36; VIG = 0.5), show that almost the entire basic variable (GER and VIG) is explained by components of seed deterioration (Table 1). Considering the direct effects of deterioration variables on germination (included in Table 1), PER (−0.68) has both the greatest effect and greatest total correlation; this, in turn, indicates a high contribution to the reduction of germination that surpasses MAL, which also had a high direct effect (−0.62). In contrast, SOD (−0.34) was the variable with the least effect as well as the lowest total correlation.

The coefficient of determination for VIG (R^2^ = 0.88) was associated with the residual effect (0.34), demonstrating that almost all of the basic variable (VIG) is explained by the seed nutrients (Table 2). This trend was not verified for the GER variable, where the coefficient of determination was low (0.23) and the effect of the residual variable, high. Considering the direct effects of nutrients on vigor (included in Table 2), Mg (0.76) has the greatest direct effect and the second highest total correlation, indicating a major contribution to increasing vigor in soybean seeds. Despite the high correlation of K with vigor (0.89), the direct effect was low (0.47), meaning that the K variable influences the main variable only indirectly, being only together with other variables. Thus, Mg indirectly contributes to the high correlation between K and VIG. These results reinforce the importance of Mg for the vigor of soybean seeds. 

### 3.6. Dissimilarity

**Figure 8:**
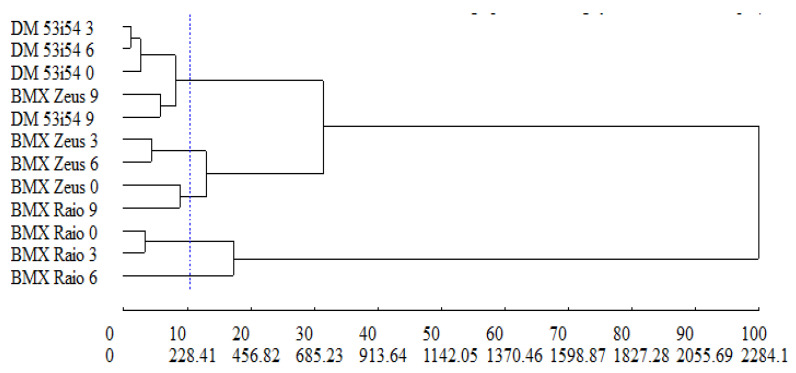
Generic dissimilarity dendrogram among three cultivars (BMX Raio, BMX Zeus, and DM 53i54) and four periods of accelerated aging (0, 3, 6, and 9 days) obtained by the UPGMA method, based on the average Mahalanobis distance matrix with superoxide dismutase, catalase, ascorbate peroxidase, lipid peroxidation, H_2_O_2_, and Maillard reaction; for cophenetic correlation, r = 0.73.

The relative contribution to dissimilarity between treatments was estimated using the Singh method [29], where the variables of greatest contribution—MAL (46.4%), CAT (29.3%), and APX (19.9%)—represented 95% of the dissimilarity between treatments. The cophenetic correlation coefficient, which estimates the representativeness of data from the dendrogram dissimilarity matrix, revealed a magnitude of 0.73, indicating that the matrix data showed satisfactory adjustment in the graphical representation presented by the dendrogram (Figure 8). The UPGMA group method allowed the formation of five distinct groups: group 1—DM 53i54 3, DM 53i54 6, DM 53i54 0, BMX Zeus 9, DM 53i54 9; group 2—BMX Raio 0, BMX Raio 3; group 3—BMX Zeus 3, BMX Zeus 6; group 4—BMX Zeus 0, BMX Raio 9; and group 5—BMX Raio 6.

In the dendrogram (Figure 8), group 1 was formed by the treatments, due to the higher MAL (average = 3.38), lower CAT (average = 0.10), lower APX (average = 0.11), and higher H_2_O_2_ (average = 10.31) in relation to the other groups. In relation to group 2, the treatments were given by the lowest MAL (average = 0.25), highest CAT (average = 0.61), highest APX (average = 0.36), and lowest H_2_O_2_ (average = 0.36). In the other groups (3, 4, and 5), treatments, for the variables mentioned above, were grouped by intermediate reactions.

### 3.7. Scanning Electron Microscopy Analysis

**Figure 9:**
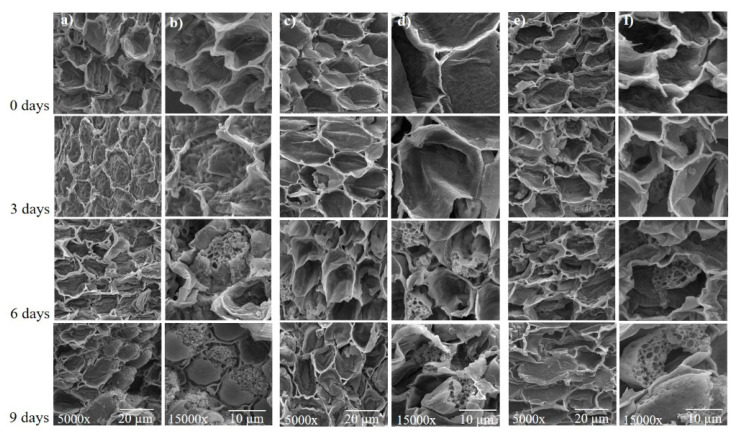
Scanning electron micrographs of the soybean seed embryonic axis of three cultivars (BMX Raio, BMX Zeus, and DM 53i54) exposed to different periods of accelerated aging (0, 3, 6, and 9 days). (**a**,**b**) BMX Raio; (**c**,**d**) BMX Zeus; (**e**,**f**) DM 53i54. The embryonic axis analyzed by cross section.

SEM analysis of the cross section of the embryonic axis of soybean seeds showed that for 0 and 3 days of accelerated aging, membrane structure remained normal; however, 6 and 9 days of accelerated aging caused significant membrane damage (Figure 9). It is possible to observe the disintegration of the membranes’ structure and the opening of pores, thus showing that a greater amount of time of accelerated aging contributes to the deterioration of soybean seeds.

## 4. Discussion

Lipid peroxidation is the main event behind seed deterioration, as the imbalance between antioxidant activity and ROS production causes the accumulation of malondialdehyde. The event behind the imbalance of ROS and antioxidant activity is the depression of catalase and ascorbate peroxidase activity, which are reduced through aging. Despite the initial hypothesis, no DNA break or oxidation were found, this also did happen with the glycation reaction (Maillard reactions), which are not impacted by aging. In the analysis of nutrients, magnesium values were, interestingly, higher in the cultivar BMX Raio RSF IPRO, which is more resilient to deterioration; this raises the question of the possible impact of magnesium in seed deterioration. In our study, we theorize that this is caused by an increase of magnesium pectates, which reduced the air space among the cells and decreased external gas diffusion in the internal tissue, leading to an accumulation of CO_2_ and causing a reduction of respiration rate. However, there is a need for new studies about the role of magnesium in seed embryo growth and deterioration.

Vigor and germination were reduced during aging, as was expected. These reductions were caused by two correlated events: antioxidant enzymes depression and lipid peroxidation (Figure 2 and Figure 3). Antioxidant enzyme depression followed an already seen pathway in rice (*Oryza sativa* L.), where SOD maintain the levels of expression and activity during accelerated aging, but with reductions in CAT and APX in both expression and activity [6]. While the results showed that BMX Raio has a higher H_2_O_2_ level than BMX Zeus, BMX Raio maintains higher levels of antioxidant enzymes, leading to a smaller increase of H_2_O_2_. Stressing that the first event in seed deterioration is antioxidant depression, it is only after this depression that the imbalance in antioxidant activity versus ROS occurs, leading to lipid deterioration [5]. The deleterious effects of lipid peroxidation were seen in the scanning electron micrographs (Figure 9), which showed that as aging progresses, the membrane started to show disruption.

The results showed that the values of H_2_O_2_ were not per se a good indicator of lipid peroxidation and the potential damage of ROS, whereas MDA served as a better indicator. This was highlighted by the fact that BMX Raio at 0 days had a higher content of H_2_O_2_ than BMX Zeus, but a smaller MDA content, as well as higher values of germination and vigor. MDA is considered to be a better indicator in this context because it is the final product of lipid peroxidation; thus, it is present only when damage occurs, while H_2_O_2_ content per se not cause damage if can be detoxifed [30]. However, not only the antioxidant enzymes can modulate the impacts of ROS, once proteins play a function in seed deterioration, where it serves as a sink for oxidative ROS during storage and germination [9]. Thus, embryos with higher protein content had a bigger sink capacity of ROS damage. However, in the present study, the percentage of embryo protein ranged from 34.9% to 38.5%, which was much higher than that found in other species such as corn (Zea mays L.) (10%) [31]. As the protein content of embryos is very high, the content of embryo protein does not provide any advantage for some specific cultivar against seed deterioration in the soybean embryonic axis.

ROS imbalance was expected to cause not only lipid peroxidation, but also genotoxic effects [5,30]. In the present study, no loss of DNA integrity was observed (Supplementary Figure S1), and DNA oxidation as accelerated aging progress. These results are caused due the method that seeds were exposed at very high humidity, and reach higher moisture content (19.1–20.3%). Since the affinity for ROS to the genetic material was higher in seeds with low moisture [32], no genetic damage was found in previous soybean embryos during accelerated aging, showing only reduction in the new mRNA; however, this was cause by the absence of sufficient energy supply and not to direct genetic damage [33].

The consumption of reserves is one of the events that happens during seed deterioration [5]. As such, the main storage products are evaluated. Despite this, protein and glucose did not show a significant reduction in the distinct periods of aging. The most important change was the decrease of total sugars, which was used to maintain the embryo. Although, as was reported above, in accelerated aging the main impact in protein expression is not genetic, but energetic [33], nevertheless, the energetic shortage presented in the embryo is not due to the runoff of reserves, but is instead caused by the physical and chemical modification of mitochondria. During aging, mitochondria membranes are disrupted in vesicles and cristae shorten [34], and a reduction in cytochrome c oxidase, malate dehydrogenase, and NADH also occurs [35]. These turn oxidative phosphorylation into a less efficient process, with a large decrease in the adenylate energy charge during germination [33], leading to the energetic collapse of the cell.

During aging, there is a depression of antioxidant enzymes, but the cause is still not clear. However, the Maillard reaction appears to be a prominent answer to the phenomenon, as the reaction of reducing sugars with proteins generated glycation end products, leading to the loss of their biological function [5]. Furthermore, this inactivation could cause depression not only in antioxidant enzymes, but also in enzymes related to DNA integrity, such as DNA ligase [36]. Although this relation is clearly demonstrated in previous studies [11,36], this is not found in the present study, because the values were stable among the periods of aging. Nevertheless, there were differences among cultivars, but the one most resilient to deterioration—BMX Raio—showed a higher value, which can indicate that the proteins of BMX Raio had more reactivity and could act as buffers for ROS damage, which does not occur in BMX Zeus and DM 53i54. However, this statement needs corroboration. Furthermore, SEM analysis showed that at 6 and 9 days, accelerated aging caused significant damage to membranes in all cultivars, contributing to the deterioration of soybean seeds. Membrane damage was also identified in soybean seeds with a water content above 30% [37], decreasing the physiological quality of seeds, since the integrity of cell membranes is very important for them to perform their functions. The loss of control of intracellular compartmentalization, with alteration in the metabolism, can cause the loss of viability of the seed [38].

**Figure 10:**
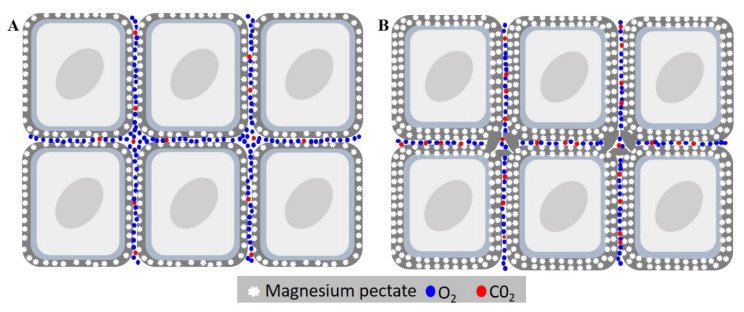
Effects of magnesium concentration in seed deterioration. (**A**) Cell of embryonic axis tissue with low magnesium content. (**B**) Cell of embryonic axis tissue with higher magnesium content. The high magnesium content causes thickening of medium lamella by the increasing of magnesium pectates, reducing gas diffusion of the internal tissue. This leads to the accumulation of CO_2_ in the tissue, reducing respiration, and leading consequently to a reduction in seed deterioration.

The nutrients of seeds have always had their role in seed deterioration underestimated, with the majority of studies only evaluating their presence in the leakage, or their content after seed imbibition [39]. However, how the content of each element impacts seed deterioration itself is still obscure. The results showed that Mg was the dissonant element, with a much higher content in the cultivar BMX Raio, which had the most resilience to seed deterioration. It is already known that in leaves, the deficiency of Mg causes an increase in MDA content by photo-oxidation as well as a reduction in CAT by photo-inactivation [40]. Since a seed is not photosynthetically active, this cannot explain the importance of Mg in the seed. Thus, we developed a hypothesis in which Mg plays a role in cellular disposal due to their function as constituent of pectates. The hypothesis is that seeds with higher Mg content had a more cohesive conformation that reduces oxygen diffusion, with the reduction in oxygen content and increase of CO_2_ inside the tissues (Figure 10) occurring the reduction of seed respiration rate and thus ROS production.

## 5. Conclusions

Lipid peroxidation is the main event behind seed deterioration; it is caused by the imbalance between antioxidant activity and ROS production, which, in turn, causes the accumulation of malondialdehyde. The event behind the imbalance of ROS and antioxidant activity is the depression of catalase and ascorbate peroxidase activity, which are reduced through aging. Despite our initial hypothesis, no DNA break or oxidation was found, the same happening vis-à-vis the glycation reaction (the Maillard reactions), which is not impacted by the aging. In the analysis of the nutrients, the values of magnesium showed an interestingly higher value in the cultivar BMX Raio RSF IPRO. As this cultivar has greater resilience to deterioration, it raises the question of the possible impact of this nutrient in seed deterioration. We theorize that this is caused by an increase of magnesium pectates, which reduce the air space among the cells, decrease external gas diffusion in the internal tissue, and lead to an accumulation of CO_2_, causing a reduction in respiration rate. However, there is a need for new studies about the role of magnesium in seed embryo growth and deterioration.

## Figures and Tables

**Table 1:** Estimates of direct and indirect effects involving the key dependent variables of germination and vigor, and the other independent explanatory variables as antioxidant enzymes, hydrogen peroxide, malondialdehyde, and Maillard reaction.

Independent Variables	SOD		CAT		APX		H_2_O_2_		MAL		MAI	
Dependent Variables	GER	VIG	GER	VIG	GER	VIG	GER	VIG	GER	VIG	GER	VIG
Direct effect	−0.34	0.11	0.37	0.79	−0.39	0.08	−0.68	−0.34	−0.62	0.3	−0.38	−0.14
Indirect effect by SOD	1	1	−0.15	0.05	−0.01	0	0.01	0	0.09	−0.03	−0.12	0.04
Indirect effect by CAT	0.16	0.35	1	1	0.3	0.63	−0.27	−0.58	−0.34	−0.72	0.33	0.69
Indirect effect by APX	−0.01	0	−0.32	0.07	1	1	0.34	−0.07	0.33	−0.07	−0.25	0.05
Indirect effect by H_2_O_2_	0.01	0.01	0.49	0.25	0.58	0.29	1	1	−0.5	−0.25	0.33	0.17
Indirect effect by MAL	0.16	−0.07	0.56	−0.27	0.51	−0.24	−0.46	0.22	1	1	0.51	−0.25
Indirect effect by MAI	−0.14	−0.05	−0.34	−0.12	−0.25	−0.09	0.19	0.07	0.32	0.12	1	1
Correlation (r)	−0.16	0.35	0.63	0.84	0.74	0.68	−0.87	−0.74	−0.72	−0.62	0.41	0.55
			GER				VIG					
Determinantion coefficient			0.87				0.75					
Effect of the residual variable			0.36				0.5					

Germination (GER), vigor (VIG), superoxide dismutase (SOD), catalase (CAT), ascorbate peroxidase (APX), malondialdehyde (MAL), and Maillard reaction (MAI).

**Table 2:** Estimates of direct and indirect effects involving the key dependent variables of germination and vigor, and the other independent explanatory variables as macronutrients.

Independent Variables	N		P	K		Ca			Mg	
Dependent Variables	GER	VIG	GER	VIG	VIG	GER		VIG	GER	VIG
Direct effect	0.29	−0.05	−0.35	0.19	0.47	−0.65		0.17	0.52	0.76
Indirect effect by N	1	1	0.04	−0.17	0.03	−0.01		0.01	−0.1	0.02
Indirect effect by Ca	−0.02	−0.02	0.13	0.06	0.07	0.15		1	0.12	0.14
Indirect effect by P	−0.05	−0.09	1	−0.13	−0.23	1		−0.6	−0.29	−0.54
Indirect effect by K	−0.11	−0.27	0.07	1	1	0.17		0.2	0.14	0.34
Indirect effect by Mg	−0.18	−0.26	0.43	0.38	0.55	0.62		0.65	1	1
Correlation (r)	−0.05	−0.69	0.29	0.36	0.89	0.29		0.42	0.44	0.72
			GER				VIG			
Determination coefficient			0.23				0.88			
Effect of the residual variable			0.88				0.34			

Germination (GER), vigor (VIG), nitrogen (N), calcium (Ca), phosphorus (P), potassium (K), and magnesium (Mg).

## Supplementary Materials

The following are available online at https://www.mdpi.com/2079-7737/9/8/186/s1, Figure S1: DNA agarose gel electrophoresis extracted from the embryonic axis of soybean from three cultivars (BMX Raio, BMX Zeus and DM 53i54) submitted to different periods of accelerated aging (0, 3, 6 and 9 days). Numbers 1 and 2–BMX Raio at 0 days; 3 and 4–BMX Raio at 3 days; 5 and 6–BMX Raio at 6 days; 7 and 8–BMX Raio at 9 days; 9 and 10–BMX Zeus at 0 days; 11 and 12–BMX Zeus at 3 days; 13 and 14–BMX Zeus at 6 days; 15 and 16–BMX Zeus at 9 days; 17 and 18–DM 53i54 at 0 days; 19 and 20–DM 53i54 at 3 days; 21 and 22–DM 53i54 at 6 days; 23 and 24–DM 53i54 at 9 days.
