# Supplementary material for: Biochemical Profile of the Soybean Seed Embryonic Axis and Its Changes during Accelerated Aging
Source: Biology (Basel). 2020 Jul 23;9(8):186. doi: 10.3390/biology9080186 (PMC7465099; doi:10.3390/biology9080186)
Supplement: Supplementary file 1 [file biology-09-00186-s001.pdf]

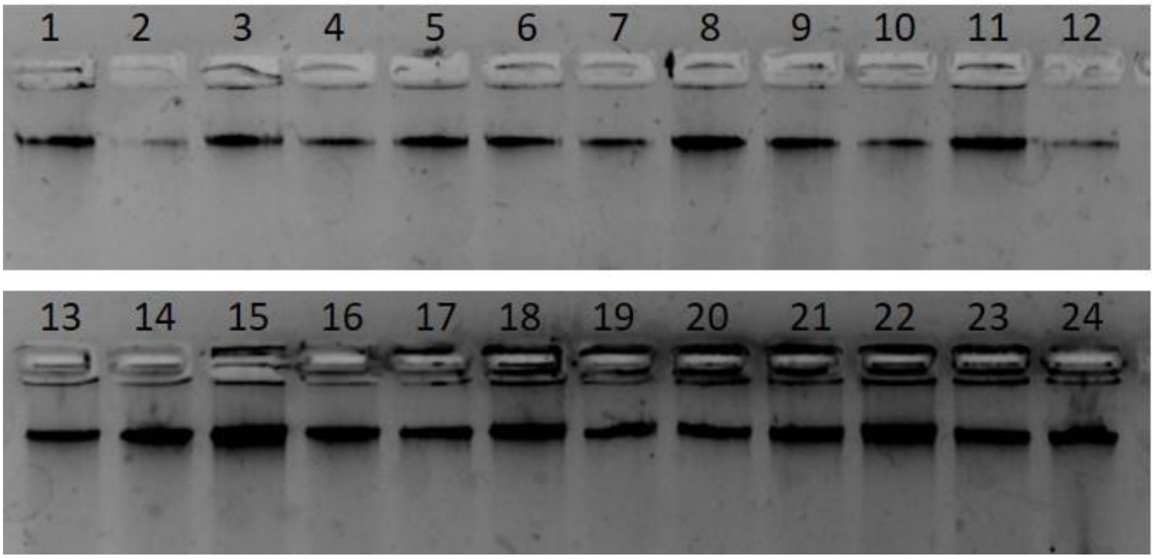

Supplementary figure 1- DNA agarose gel electrophoresis extracted from the embryonic axis of soybean from three cultivars (BMX Raio, BMX Zeus and DM 53i54) submitted to different periods of accelerated aging (0, 3, 6 and 9 days). Numbers 1 and 2 – BMX Raio at 0 days; 3 and 4 – BMX Raio at 3 days; 5 and 6 – BMX Raio at 6 days; 7 and 8 – BMX Raio at 9 days; 9 and 10 – BMX Zeus at 0 days; 11 and 12 – BMX Zeus at 3 days; 13 and 14 – BMX Zeus at 6 days; 15 and 16 – BMX Zeus at 9 days; 17 and 18 – DM 53i54 at 0 days; 19 and 20 – DM 53i54 at 3 days; 21 and 22 – DM 53i54 at 6 days; 23 and 24 – DM 53i54 at 9 days.
